# Endogenously-Activated Ultrasmall-in-Nano Therapeutics: Assessment on 3D Head and Neck Squamous Cell Carcinomas

**DOI:** 10.3390/cancers12051063

**Published:** 2020-04-25

**Authors:** Melissa Santi, Ana Katrina Mapanao, Domenico Cassano, Ylea Vlamidis, Valentina Cappello, Valerio Voliani

**Affiliations:** 1 Center for Nanotechnology Innovation@NEST, Istituto Italiano di Tecnologia, Piazza San Silvestro, 12-56126 Pisa, Italy; katrina.mapanao@sns.it (A.K.M.); domenico.cassano@sns.it (D.C.); ylea.vlamidis@iit.it (Y.V.); valentina.cappello@iit.it (V.C.); 2 NEST-Scuola Normale Superiore, Piazza San Silvestro, 12-56126 Pisa, Italy

**Keywords:** cancer, cisplatin, 3D models, nanomaterials, theranostics

## Abstract

Negative or positive HPV-associated Head and Neck Squamous Cell Carcinomas (HNSCCs) are high recurrence neoplasms usually resulting in a poor prognosis, mainly due to metastasis formation. Despite the low overall patient survival rate and the severe side effects, the treatment of choice is still cisplatin-based chemotherapy. Here, we report a straightforward protocol for the production of high throughput 3D models of negative or positive HPV-associated HNSCCs, together with their employment in the therapeutic evaluation of gold ultrasmall-in-nano architectures comprising an endogenously-activatable cisplatin prodrug. Beyond enhancing the biosafety of cisplatin, our approach paves the way for the establishment of synergistic co-therapies for HNSCCs based on excretable noble metals.

## 1. Introduction

Head and Neck Squamous Cell Carcinoma (HNSCC) is a complex group of malignancies that affect different body sites, among which the oral cavity, nasopharynx, oropharynx, larynx, and salivary glands. HNSCC is one of the most common cancer types with 53,000 new cases estimated in 2019 only in the United States [1], and it is mainly caused by extensive consumption of tobacco and alcohol [2]. Usually, HNSCCs are characterized by mutation of the p53 and p16 genes and often results in a poor prognosis and a high risk of recurrence/metastasis (R/M HNSCC) [3]. Recently, a new subset of HNSCC with peculiar biological and clinical features was associated with Human Papilloma Virus (HPV) infection [4,5]. HPV-16 and HPV-18 subtypes have the highest incidence and induce malignancies both in HNSCC and cervical cancers [6]. They encode for E6 and E7 viral proteins, which induce dysregulation of the cell cycle interacting with p53 and pRb, respectively [7]. HPV-positive cancer patients usually show better survival rates with respect to HPV-negative counterparts with an improved overall survival, but the molecular mechanisms of these responses are still debated [8,9]. When possible, patients are primarily subjected to local surgery and radiotherapy, followed by chemotherapy, which instead is the only treatment in the case of R/M HNSCC [10]. Single or combined cisplatin-based therapies are the principal treatments for HNSCCs neoplasms and their sensitivity is similar for ±HPV-associated conditions, even if their sub-optimal efficacy is limited by associated severe systemic toxicities, such as nephrotoxicity, neurotoxicity, ototoxicity, and emesis [11]. In this context, Pt(IV) complexes provide an attractive alternative to Pt(II) compounds because their inertness results in fewer side effects, decreased drug inactivation and a longer half-life in the bloodstream [12]. Pt(IV) prodrugs are hidden forms of cisplatin, whose production is triggered by endogenous thiols, such as glutathione, which lead to reductive elimination of axial ligands [13]. The inclusion of Pt(IV) prodrugs in biodegradable nanomaterials enhances the specificity of cisplatin due to the defined localization and endogenously-double-controlled release of the active drug [14]. Beyond drug delivery, the conjugation of prodrugs with nanomaterials, and in particular to noble metals, is essential for the development of innovative or combined/enhanced treatments [15,16]. For example, nanostructured noble metals can be exploited for their peculiar behaviors in photothermal therapy [17], radiation therapy enhancement [18], and in imaging applications as an ultrasound or photoacoustic [19,20]. On the other hand, despite the massive efforts, co-treatments based on noble metal nanomaterials are still at a preclinical stage, due to the body persistence issue [16,21]. Hitherward, our group introduced biodegradable/excretable nano-architecture (NAs) composed of silica nanocapsules comprising plasmonic ultrasmall noble metal nanoparticles, that may again bring inorganic nanomaterials to the forefront of clinical applications [21,22]. Indeed, NAs jointly combine the behaviors of plasmonic nanomaterials with a good clearance rate of the building blocks [23,24]. Besides demonstrating their massive production and protocol reproducibility [15,25], we have already confirmed their biosafety [24,26], assessed the biokinetics [23,24], and currently identified some potential applications [14,17,19,20]. Here, NAs comprising a Pt(IV) prodrug (NAs-cisPt) were produced and the therapeutic efficacy fully assessed on customized 3D models of two ±HPV-associated HNSCCs cell lines: SCC-25 (HPV-negative) and UPCI:SCC-154 (HPV-positive). 3D models better represent the complexity of neoplasms with respect to 2D cell cultures and the results are useful for the progress of preclinical oncological investigations and the translation of nanomaterials to clinics [27,28]. Different types of 3D models are available and can be used depending on the investigations that have to be carried out: cells can be induced to form tridimensional structures or can be embedded in a specific scaffold that mimics extracellular matrix (e.g., Matrigel) [29,30]. Some models can also present more complex structures, like chorioallontoic membranes (CAMs), for pharmacological studies that also include the vascular component [31]. Among all, tumor spheroids represent the best option for the study of nanoparticles efficacy, since they are composed of different populations of quiescent/necrotic and proliferative cells, comprising gradients of nutrients and oxygen and at the same time are cheap and easy to handle [32]. In particular, they allow for obtaining reliable information on the efficiency of nanomaterials and internalization mechanisms, taking also into account cell–cell and cell–extracellular matrix (ECM) interactions [33,34]. Overall, our findings are a significant step forward toward the translation of novel and efficient noble metal-based co-treatments for patients.

## 2. Results and Discussion

**Figure 1:**
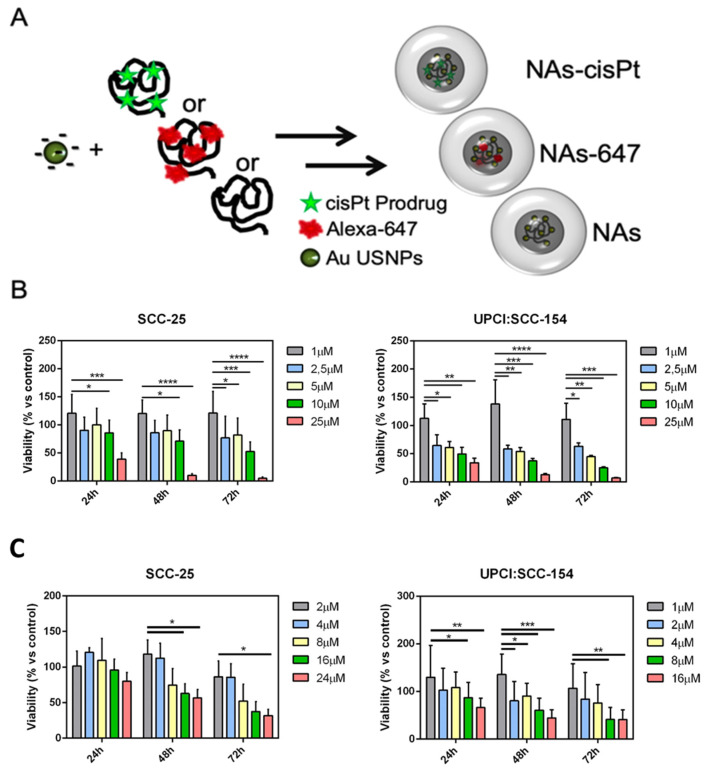
Structures of NAs and cytotoxic effect of cisplatin-loaded nano-architectures (NAs) against SCC-25 and UPCI:SCC-154. (**A**) Cartoon depicting the production protocol of the three kinds of NAs employed in this work. Each cell line was treated with increasing concentrations of (**B**) free cisplatin or (**C**) NAs-cisPt, for 2 h at 37 °C and 5% of CO_2_. Cells viability was measured at 24 h, 48 h and 72 h after treatment, and data were normalized to the viability of control cells (treated only with medium). Concentrations in the graph correspond to the active cisplatin comprised in NAs-cisPt. Results are the average of three independent experiments and error bars state the standard deviation. Two-way ANOVA Dunnett’s test for (**B**) vs. 1 µM, (* *p* ≤ 0.05, ** *p* ≤ 0.01, *** *p* ≤ 0.001 and **** *p* ≤ 0.0001) and for (**C**) vs. 2 µM or 1 µM for SCC-25 and UPCI:SCC-154 respectively, (* *p* < 0.05, ** *p* ≤ 0.01 and *** *p* ≤ 0.001).

In this work, we employed NAs internally-labelled with Alexa Fluor-647 (NAs-647) or internally-loaded with a cisplatin prodrug (NAs-cisPt) to investigate, respectively, the internalization behaviors and the cytotoxicity trends in 2D and 3D SCC-25 and UPCI:SCC-154. NAs-cisPt were synthesized following the protocol employed for the production of standard passionfruit-like nanoarchitectures (NAs) with slight modifications, as described in our previous works (Figure 1A) [14,19]. Briefly, ultrasmall gold nanoparticles (USNPs) were synthesized by fast reduction of an aqueous solution of chloroauric acid by sodium borohydride in the presence of poly(sodium 4-styrene sulfonate) (PSS). Then, USNPs were mixed with a Pt(IV) prodrug-modified poly-L-lysine aqueous solution in order to form controlled aggregates. Finally, the polymer templates were employed to compose a 20-nm-thick silica shell by using a modified Stöber process [22]. Resulting NAs-cisPt contained 4.9% and 1.4% (*w/w*) of, respectively, gold and platinum (meaning 21 µg/mg of cisPt/nano-architectures) on the total freeze-dried sample weight, quantified by Inductively Coupled Plasma Mass Spectrometry (ICP-MS) analysis. A full set of characterizations was performed to check the products and the reproducibility of the protocol (Figure S1 and Table S1). Standard NAs (silica capsule comprising only USNPs) and Alexa Fluor 647-modified NAs (NAs-647) were produced and routinely characterized as reported in [14,23]. The general structure of all nano-architectures is reported in Figure 1A and Figure S2. NAs-647 were firstly employed in order to evaluate by confocal microscopy their internalization behaviors in ±HPV-associated HNSCCs. The nano-architectures were efficiently internalized in SCC-25 and UPCI:SCC-154 cell lines by endocytosis, as confirmed by the characteristic punctate signals arising from nanoparticles inside endocytic vesicles and lysosomes (Figure S3). This result was not surprising, as both bare and labelled NAs have generally demonstrated good internalization behaviors in 2D and in 3D cell cultures of MIA PaCa-2, a model of pancreatic ductal adenocarcinoma (PDAC) [14,34]. It is worth remembering that the internalization features of the three versions of nano-architectures (NAs, NAs-cisPt, and NAs-647) are expected to be similar because there are no differences in their external surfaces, as supported as well by comparable zeta potential surface charge (Table S1). Indeed, NAs-cisPt were efficiently internalized in both ±HPV-associated HNSCCs, inducing cell death with a differential sensitivity (Figure 1C). Surprisingly, UPCI:SCC-154 resulted in slightly more sensitivity to cisplatin with respect to SCC-25, and this finding was also consistent with the IC_50_ values calculated by WST-8 viability assay (8.2 ± 0.1 µM and 3.5 ± 0.1 µM, respectively for -HPV and +HPV HNSCC) associated with the effect of the drug alone (Figure 1B and Table S2). Indeed, several works have discussed that ±HPV-associated HNSCCs do not have a different sensitivity to cisPt rather than radiotherapy [35]. It is worth noticing that, other than cisplatin alone, NAs-cisPt produced significant effects on cell lines (Figure 1C) only after 48 h from the incubation timepoint. This behavior is related to the degradation timeframe of the nano-architectures (24 h) followed by the conversion of the prodrug to its active form, that is able to penetrate the nucleus and binds the DNA, inducing therapeutic action [14].

Additional viability assays with standard NAs were performed to confirm that the observed cytotoxic effect of NAs-cisPt was effectively induced by released cisplatin. The amount of nano-architectures employed to treat cells was normalized on gold, i.e., the amount of gold (and thus of nano-architectures) employed was similar for each condition with both NAs and NAs-cisPt (Figure S4). As already demonstrated on zebrafish and murine models, the biosafety of standard NAs was also confirmed for these cell lines [23,24,26].

Multicellular tumor spheroids (MCTSs) are high-throughput and accessible models that well-represent neoplasms [34]. They possess more complex biological features compared to monolayer cell cultures and allow the investigation of important features such as cell-cell and cell-extracellular matrix interactions. These behaviors play a fundamental role on the effective therapeutic action of nanostructured materials. Furthermore, although investigations on animal models remain inevitably important in preclinical studies, more strict directives are now being implemented (such as the European Directive 2010/63/EU) to encourage the use of alternative biological systems in order to promote a more responsible employment of animals in research (3R’s concept: reduce, refine, and replace) [36]. 3D models of ±HPV-associated HNSCCs were produced by a slightly modified protocol of the hanging drops approach from Foty et al. [37]. A cell suspension containing 1 × 10^6^ cells/mL was used to produce drops on the lid of a petri dish. A volume of 10 µl or 20 µl was used, respectively for SCC-25 and UPCI:SCC-154, to prepare drops. After 3 days, a sheet of cells was formed in each drop that was transferred to a suspension plate in order to induce cell aggregation by orbital shaking (Figure S6). The spheroids produced by our standardized protocol show a diameter of 200–400 µm for both cell lines, and their structure was evaluated by transmission electron microscopy (Figure S5).

Firstly, with the ultrastructural analysis we were able to confirm the presence of the virus in UPCI:SCC-154 cell cytoplasm (Figure S7A). Both cell monolayers showed a high number of microvilli that were also able to form tight junctions in the corresponding three-dimensional structures. More specifically, SCC-25 form compact structures with an external layer of dividing cells and an internal layer with necrotic cells. Here, the presence of junctions was more pronounced as evidenced by the inset in Figure S5C. On the other hand, UPCI:SCC-154 formed less compact spheroids that showed an internal empty cavity. In particular, cells were arranged in different layers like a sandwich. Four different layers were identified from TEM analysis: starting from the periphery the first two layers were formed by viable cells, followed by one internal necrotic portion and a final layer composed again of living cells, as demonstrated from the poor staining of the third layer in Figure S7B. Interestingly, a very dense matrix surrounds cells of both 3D models.

**Figure 2:**
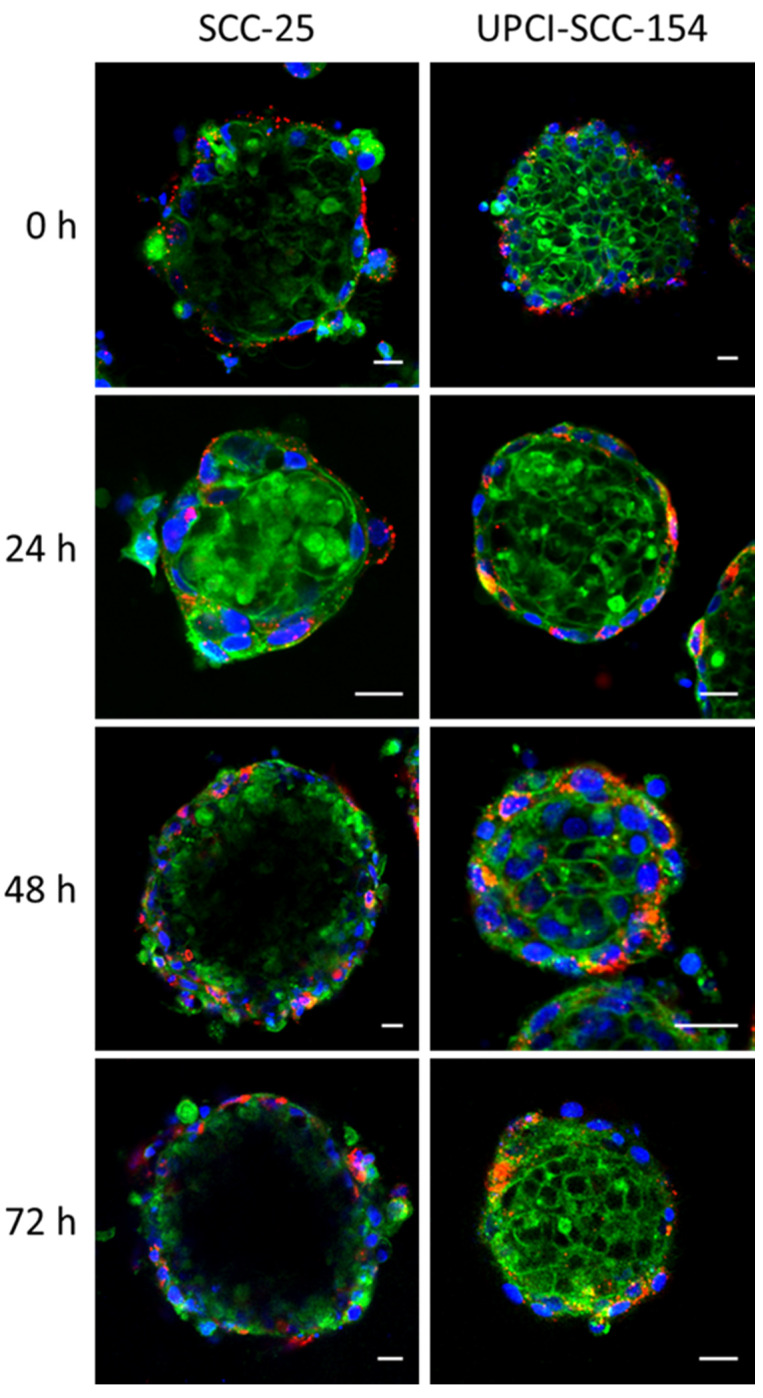
Confocal analysis of nanoparticles internalization in 3D models of HNSCC cell lines. SCC-25 and UPCI:SCC-154 spheroids were incubated with fluorescently labeled nanoparticles (NAs-647). A cell membrane marker (CellMask Green-488, Life Technologies) and Hoechst 33342 (Sigma) for nuclei were used. Spheroids were washed twice with PBS and imaged by confocal microscopy. Scale bar: 100 µm.

The complex three-dimensional structure of the spheroids, highlighted by the ultrastructural analysis, strongly supports reliable investigations of the effects of novel nanomaterials on ±HPV-associated HNSCCs. Spheroids of both ±HPV-associated HNSCCs were treated with NAs-647 for 2 h and the nanoparticles internalization process was followed for 72 h by confocal microscopy (Figure 2). Immediately after incubation and for the first 24 h, the nano-architectures showed the typical punctate pattern due to endocytic internalization. It is worth noting that despite the surrounding matrix and the complexity of the system, the nano-architectures were efficiently internalized in both the models. Indeed, as demonstrated by the ultrastructural analysis, spheroids usually present different layers of cells as a consequence of gradients of oxygen and nutrients. In particular, only the external layer is composed by actively dividing cells, thus the ability of our nanostructures to reach the first cell layer is a significant result. Furthermore, the fluorescence signal was spread inside cells after 48 h, confirming the degradation of the silica shell and the release of the dye in the cytosol. Finally, we would like to underline that confocal microscopy is a good technique that allows preliminary results about qualitative nanoparticles distribution in cells to be obtained. Unfortunately, the major limit of this optical method is related to the single focal plane imaging. A more quantitative analysis on accurate nanoparticles internalization is reported later in this work.

**Figure 3:**
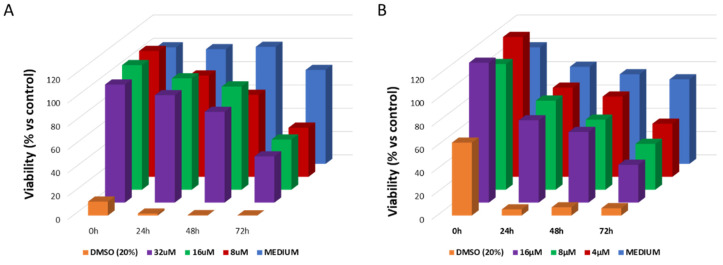
Cytotoxic effect of cisplatin-loaded nanoparticles on 3D models of HNSCCs. Cells of (**A**) SCC-25 and (**B**) UPCI:SCC-154 were treated with increasing concentration of NAs-CisPt. Spheroids without any treatments (MEDIUM) and treated with a solution of Dimethyl Sulfoxide 20% *v/v* (DMSO) were used as negative and positive controls, respectively. Cytotoxicity was monitored from 0 h to 72 h using the CellTiter-Glo^®^ 3D Cell Viability Assay. Results are the average of three independent experiments (error bars are resumed in Tables S3 and S4). Two-way ANOVA Dunnett’s test vs. MEDIUM, no significant statistical differences were found.

Finally, spheroids of the two cell lines were treated with increasing concentration of NAs-cisPt (corresponding to increasing concentration of cisplatin) for 2 h, in order to evaluate their therapeutic effect. The viability was calculated using the CellTiter-Glo^®^ 3D Cell Viability Assay, until 72 h after treatment (Figure 3). We preferred to use a commercial test because other approaches like size changes were useless with these two cell lines. Indeed, we saw that in general, the spheroids obtained from SCC-25 and UPCI:SCC-154 cell lines do not change in size once formed. This is probably due to the presence of the extracellular matrix layer, that we confirmed by TEM analysis. In this regard, we also observed that these spheroids could not be used for more than two weeks because cells continue to divide until they reach a saturation level (in which they start to die) without the spheroids demonstrating substantial change in size.

In general, NAs-cisPt affected the spheroids viability for both cell lines, confirming the distribution of the nano-architectures in the 3D models. Interestingly, viability of SCC-25 was almost constant for all the tested concentrations. This trend is probably associated with a plateau in the internalization rate of the nano-architectures. On the other hand, UPCI:SCC-154 demonstrated a dose-dependent viability trend, and this behavior may be associated to both the different density of cells in the spheroids and the cellular adhesion. As for 2D cultured cells, a slightly increased sensitivity of UPCI:SCC-154 to cisplatin with respect to SCC-25 was observed. In order to confirm that this effect was not associated to a different endocytosis rate in 3D models, the gold content inside spheroids was quantified by ICP-MS after 2 h incubation with the same amount of NAs-cisPt. As expected, the amount of gold was almost similar in both samples (0.29 ± 0.0013 and 0.27 ± 0.012 µg/µg gold/lysate, respectively for UPCI:SCC-154 and SCC-25). Thus, the effect may be associated with a potential different release trend of the drug in the cell lines. These results suggest that NAs-cisPt are efficiently internalized in these 3D cell culture models and induce an effective cell death with a reduced off-target action as a result of the double-controlled endogenous release. It is also worth noting that due to the presence of plasmonic USNPs, NAs are promising nanoplatforms for combined therapies and for theranostics development.

## 3. Materials and Methods

All chemicals were purchased from Sigma-Aldrich (St. Louis, MO, USA) unless otherwise specified. All chemicals were used as received.

### 3.1. Synthesis of Standard Nano-Architectures

*Synthesis of gold nanoparticles.* Ultrasmall gold nanoparticles with a diameter of approximately 3 nm were prepared according to the following procedure. To 20 mL of milliQ water, 10 µL of poly(sodium 4-styrene sulfonate) (70 kDa, 30% aqueous solution, PSS) and 200 µL of HAuCl_4_ aqueous solution (10 mg/mL) were added. During vigorous stirring, 200 µL of sodium borohydride (8 mg/mL in milliQ water) was added quickly, and the mixture was vigorously stirred for 2 min. After the addition of NaBH_4_, the solution underwent some color changes until it became a brilliant orange. Before its use, the solution was aged for 10 min and employed without further purification.

*Synthesis of gold nanoparticle arrays w/o dyes.* 20 mL of gold nanoparticle solution was added to a 50 mL round bottomed flask followed by 200 µL water solution of poly(L-lysine) hydrobromide 15–30 kDa (PL, 20 mg/mL or the same amount of a dye-modified poly-Lysine), and the mixture was allowed to stir for 20 min at room temperature. The as-synthesized gold aggregates were collected by centrifugation (13,400 rpm for 3 min), suspended in 2 mL of milliQ water and sonicated for a maximum of 4 min.

*Synthesis of Dye-Modified Poly(L-Lysine)–PLa.* Poly(L-lysine) hydrobromide 15–30 kDa (1.5 mg) was dissolved in PBS (780 µL) and AlexaFluor 647-NHS ester (200 µg, Invitrogen, Carlsbad, CA, USA) was added to the solution. The reaction mixture was kept under stirring overnight at RT, and it was used without further purification.

*Synthesis of nano-architectures (NAs).* 70 mL of absolute ethanol followed by 2.4 mL of ammonium hydroxide solution (30% in water), and 40 µL of tetraethyl orthosilicate (TEOS, 98%) were added in two 50 mL plastic Falcon tubes. Then, 2 mL of the gold nanoparticle arrays previously prepared were added to the Falcon (1 mL each) and the solution was allowed to gently shake for a further 3 h. The as-synthesized NAs were collected by 30 min centrifugation at 4000 rpm, washed twice with ethanol to remove unreacted precursors and suspended in 1 mL of ethanol. A short spin centrifugation was employed in order to separate the structures over 150 nm from the supernatant, which was recovered as a pink-iridescent solution. The solution containing about 1.5 mg of NAs was stored at −20 °C until use. It remains usually stable for at least one year. Product recovery: (i) 2 min centrifugation at 13,400 rpm, (ii) remove the colorless supernatant, and (iii) add the solvent of interest. The solubility of NAs in water, buffers, and physiological fluids is tested for up to 60 mg/mL.

### 3.2. Synthesis of Drug Loaded Nano-Architectures

*Synthesis of c,t,c-[PtCl_2_(OH)_2_(NH3)_2_].* The method used was based on the one previously described by Hall et al. [38] Cis-[PtCl_2_(NH_3_)_2_] (0.40 g, 1.33 mmol) was suspended in milliQ water (10 mL) and H_2_O_2_ 30% (*w/v*) (14 mL, tenfold excess) was added. The mixture was stirred for 1 h at 50 °C. Then it was cooled to 0 °C and saturated water solution of NaCl (10 mL) was added. The resultant pale yellow powder was collected by filtration and washed with cold water, ethanol and diethyl ether, and dried in a vacuum pump, yielding c,t,c-[PtCl_2_(OH)_2_(NH_3_)_2_] (223 mg, 0.67 mmol, 50%).

*Synthesis of c,t,c-[PtCl_2_(NH_3_)_2_(OH)(O_2_CCH_2_CH_2_CO_2_H)].* The method used was based on that previously described by Dhar et al. [12] To a solution of c,t,c-[PtCl_2_(OH)_2_(NH_3_)_2_] (0.2 g, 0.6 mmol) in anhydrous dimethyl sulfoxide (DMSO, 16 mL) was added succinic anhydride (0.06 g, 0.6 mmol) and the reaction mixture was stirred at room temperature for 12 h. The solution was freeze-dried and acetone (10 mL) was added to precipitate a light yellow solid, which was collected by filtration and washed several times with acetone, diethyl ether, and then dried in a vacuum pump, yielding c,t,c-[PtCl_2_(NH_3_)_2_(OH)(O_2_CCH_2_CH_2_CO_2_H)] (0.16 g, 0.37 mmol, 62%). 1H NMR (DMSO-d6) 6.18–5.66 (m, 6H), 2.44–2.33 (m, 4H). 13C NMR (DMSO-d6) 31.16; 31.74; 174.55; 180.17.

*Synthesis of Drug-Modified Poly(L-Lysine)–PLp.* c,t,c-[PtCl_2_(NH_3_)_2_(OH)(O_2_CCH_2_CH_2_CO_2_H)] (0.5 mg) was dissolved in PBS buffer (400 µL) and mixed with of freshly made EDC/NHS (40 µL, 0.21 M) milliQ water solution (EDC: N-(3-dimethylaminopropyl)-N′-ethylcarbodiimide hydrochloride, NHS: N-Hydroxysuccinimide). After 10 min stirring at room temperature, poly(L-lysine) hydrobromide 15–30 kDa (75 µL, 20 mg/mL milliQ solution) was added to the reaction mixture and the resulting solution was stirred overnight at room temperature. The modified poly(L-lysine) was collected and washed three times with PBS buffer by Amicon 10 K filter units, and then dissolved in PBS buffer (800 µL).

*Synthesis of cisplatin loaded nano-architectures (NAs-cisPt).* The protocol used is the same as that of standard nanoparticles but in this case, we used drug-modified poly(L-lysine) to form gold nanoparticles arrays.

### 3.3. Dynamic Light Scattering (DLS) Measurements

Measurements by DLS were performed at 37 °C in a 1-mL polypropylene cuvette on a Zetasizer nano-ZS DLS (Malvern Instruments, Malvern, United Kingdom) following the manufacturer’s instructions. PBS solutions of nanomaterials were analyzed with a single scattering angle of 90°. Each value reported is the average of five consecutive measurements.

### 3.4. Inductively Coupled Plasma Mass Spectrometry (ICP-MS) Analysis

The amount of gold and platinum inside nanoparticles were measured by ICP-MS analysis. 10 µl of samples was digested in 200 µl of aqua regia at 80–100 °C for 4 h. Then the solution was diluted to 2 mL and analyzed by ICP-MS (7700 series ICP-MS, Agilent Technologies, Santa Clara, CA, USA). To measure the amount of gold in spheroids, each sample was firstly lysated with 100 µl of RIPA buffer for 1 h and the protein content was measured by Bradford assay. Then, samples were digested with 200 µL of aqua regia at 80–100 °C and diluted to 2 mL for ICP-MS analysis. The gold content was expressed as µg of gold/µg of lysate.

### 3.5. UV–Vis Spectrophotometry

The absorption spectra were obtained using a double-beam Jasco V-550 spectrophotometer. The samples in PBS (1×) were placed in quartz cuvettes with a 1.5 mm path length.

### 3.6. Transmission Electron Microscopy

The TEM observations of particles and cells were performed in ZEISS Libra 120 PLUS operating at 120 KV and equipped with an In-column Omega filter. The colloidal solutions of the nanoparticles (5 μL) were deposited to a 300-mesh of carbon coated copper grids.

### 3.7. Ultrastructure Analysis of 2D and 3D cultures of SCC-25 and UPCI-SCC-154

2D cell culture or spheroids in suspension have been fixed in 1.5% glutaraldehyde in sodium cacodylate buffer (0.1 M pH 7.4) for 1 h at room temperature and then treated for resin embedding. Briefly, scraped cells or recovered spheroids were kept in a new fixative solution (overnight at 4 °C), then the samples were postfixed 1 h (1% OsO_4_ plus 1% K_3_Fe(CN)_6_ sodium cacodylate buffer; 0.1 M pH 7.4), and stained with 3% solution of uranyl acetate in 20% ethanol. Finally, they were dehydrated in a growing series of ethanol gradient and embedded in epoxy resin (Epon 812, Electron Microscopy Science, Hatfield, PA, USA). Polymerization was performed for 48 h at 60 °C. In order to perform ultrastructural analysis, 90 nm sections of the treated samples were cut by using UC7 (Leica Microsystems, Vienna, Austria) and collected on 300 mesh copper grids (EMS).

### 3.8. Cell Culture

Human squamous cell carcinoma SCC-25 and UPCI-SCC-154 were purchased from the American Type Culture Collection (ATCC). SCC-25 were maintained in a complete growth medium composed by a 1:1 mixture of Dulbecco’s modified Eagle’s medium and Ham’s F12 medium while UPCI-SCC-154 were growth in Dulbecco’s modified Eagle medium (DMEM) from Invitrogen (Carlsbad, CA, USA). Both growth mediums were supplemented with 10% fetal bovine serum (FBS), 4 mM L-glutamine, 1 mM sodium pyruvate, 100 U/mL penicillin, and 100 mg/mL streptomycin (Invitrogen). SCC-25 medium was also supplemented with 400 ng/mL of hydrocortisone. Cells were maintained at 37 °C in a humidified 5% CO2 atmosphere. For 3D structures, we used a modified protocol from Foty et al. [37] Briefly, cells were harvested and centrifuged for 5 min at 1200 rpm and then resuspended in fresh medium, counted and the suspension was adjusted to a final concentration of 1 × 10^6^ cells/mL. Afterwards, 10 µL (for SCC-25) or 20 µL (for UPCI-SCC-154) of cells were placed on the lid of a 100-mm cell culture dish that was flipped into the chamber containing 10 mL of PBS. Cells were left to settle into the drops until they formed a sheet and then were transferred to a 100 mm suspension culture dish after 3 days. Finally, cell aggregates were placed inside a CO_2_ incubator with an orbital shaker (70 rpm) for 24 h to induce the formation of the proper spherical shape.

### 3.9. Confocal Microscopy

Cells were seeded 24 h before the experiments into a glass-bottom Petri dish (WillCo-dish GWSt-3522) to reach 80–90% of confluence. Incubation of nanoparticles (maximum 30 µg) was performed for 30 min at 37 °C, 5% CO_2_ in complete medium (DMEM) with 10% FBS (total volume of 1 mL). After incubation, cells were washed twice with PBS, fresh medium was added and the samples were analyzed by confocal microscopy. For experiments with 3D models, 3–4 spheroids for each experimental condition were imaged using 2D and 3D samples, which were imaged on an Olympus FV1000 inverted confocal laser scanning microscope equipped with a thermostat chamber set at 37 °C and 5% CO_2_. The lasers for excitation were 405, 488 and 633 nm. All images were analyzed using Fiji-ImageJ software version 1.51 s.

### 3.10. Viability Assay on 2D Cell Culture

The cytotoxicity of cisplatin, NAs and NAs-cisPt was evaluated by using a tetrazolium salt, 2-(2-methoxy-4-nitrophenyl)-3-(4-nitrophenyl)-5-(2,4-disulfophenyl)-2H tetrazolium, and monosodium salt (WST-8) assay. SCC-25 and UPCI-SCC-154 cells (1 × 10^4^ cells per well) were seeded in 96 well plates. After being cultured for 24 h, the cells were incubated with a 2% serum-containing medium in the presence of free drug or nanoparticles at increasing concentration in medium (100 μL) for 30 min at 37 °C. After incubation, the medium was removed and cells were washed twice with PBS and kept in fresh medium. For each experimental time point cells were incubated with WST-8 reagent (100 μL) and 2% serum-containing medium (90 μL) for 2 h. Absorbance (450 nm) was measured using a microplate reader (Glomax Discovery, Promega, Madison, WI, USA). The percentage of cell viability was determined by comparing drug-treated cells with the untreated cells (100% viability). Data represent the average of three independent experiments. Error bars represent the SD from three independent experiments.

### 3.11. Viability Assay on 3D Cell Models

Viability of 3D spheroids was evaluated using the CellTiter-Glo^®^ 3D Cell Viability Assay (Promega, Milan, Italy). Treated or untreated spheroids for each time point were transferred separately from a round-bottom 96-well plate to a white 96-well plate for luminescence measurements with 100 µL of medium. Then 100 µL of CellTiter-Glo^®^ 3D reagent was added to each well, the plate was shaken for 5 min and the luminescence signal was recorded after 25 min of incubation with a microplate reader (Glomax Discovery, Promega, Madison, WI, USA). Cell viability was determined with respect to the viability of spheroids maintained in complete medium without any other treatments.

## 4. Conclusions

In summary, we have described a robust protocol for the production of fully characterized 3D ±HPV-associated HNSCCs models together with their employment in nanomaterials efficacy evaluation. Biodegradable and excretable NAs comprising cisPt prodrug can be efficiently internalized in 2D and 3D cell culture and have demonstrated an endogenously-double-controlled therapeutic activity, showing interesting toxicity trends. In particular, we evidenced a more than 50% decrease of cell viability of our 3D models in both cell lines 72 h after treatment. Moreover, the exploitation of the Pt(IV) complexes can limit the systemic toxicity of cisplatin paving the way for more efficient management of HNSCCs. Remarkably, excretable gold USNPs comprised in NAs-cisPt also enable the potential integration of noble metal-driven imaging and treatments, supporting the development of safe co-treatments for HNSCCs.

## Supplementary Materials

The following are available online at https://www.mdpi.com/2072-6694/12/5/1063/s1, Figure S1: Physical-chemical characterization of NAs-CisPt, Figure S2: General structure of nano-architectures, Figure S3: Internalization of NAs-647 in HNSCC-25 and UPCI-SCC-154 cell lines, Figure S4: Cytotoxic effect of Cisplatin against HNSCC-25 and UPCI-SCC-154, Figure S5: Cytotoxic effect of standard gold nanoparticles against SCC-25 and UPCI-SCC-154, Figure S6: Bright field images of SCC-25 and UPCI-SCC-154 spheroids, Figure S7: Ultrastructural characterization of different layers of 3D UPCI-SCC-154, Table S1: Size and zeta potential of NAs, NAs-647 and NAs-cisPt, Table S2: IC50 measurements for SCC-25 and UPCI-SCC-154 measured 24 h after treatment with free or nanoparticles-loaded cisplatin, Table S3: Viability of SCC-25 spheroids after treatment with NAs-cisPt, Table S4: Viability of UPCI-SCC-154 spheroids after treatment with NAs-cisPt.
