# Supplementary material for: Endogenously-Activated Ultrasmall-in-Nano Therapeutics: Assessment on 3D Head and Neck Squamous Cell Carcinomas
Source: Cancers (Basel). 2020 Apr 25;12(5):1063. doi: 10.3390/cancers12051063 (PMC7281743; doi:10.3390/cancers12051063)
Supplement: Supplementary file 1 [file cancers-12-01063-s001.pdf]

Supplementary Materials

Endogenously-Activated Ultrasmall-in-Nano Therapeutics: Assessment on 3D Head and Neck Squamous Cell Carcinomas

Melissa Santi, Ana Katrina Mapanao, Domenico Cassano, Ylea Vlamidis, Valentina Cappello and Valerio Voliani

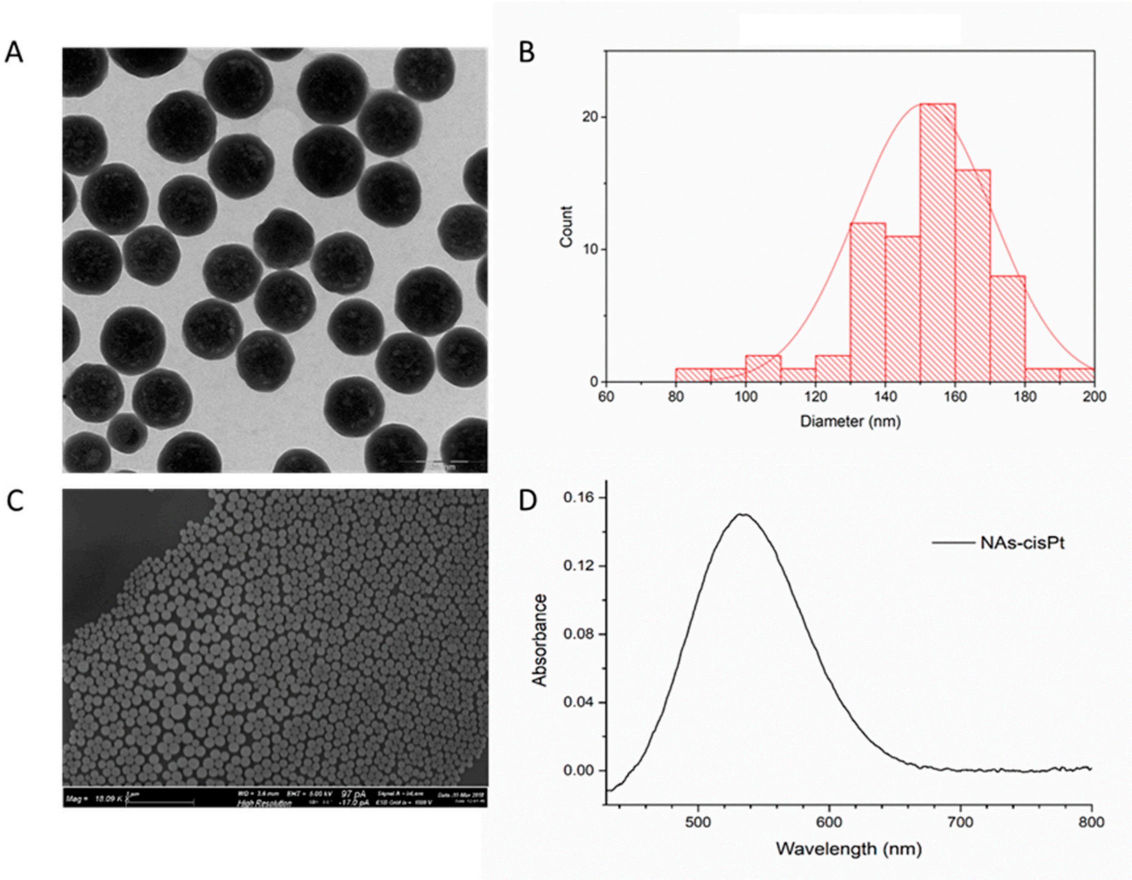

Figure S1. Physical-chemical characterization of NAs-cisPt. **A** TEM images of NAs-cisPt. Scale bar: 200 nm. **B** Size histogram of NAs-cisPt made on at least 100 NAs observed by TEM. **C** SEM images of NAs-cisPt. Scale bar: 1 μm. **D** Background subtracted UV/vis absorbance spectrum of and NAs-cisPt.

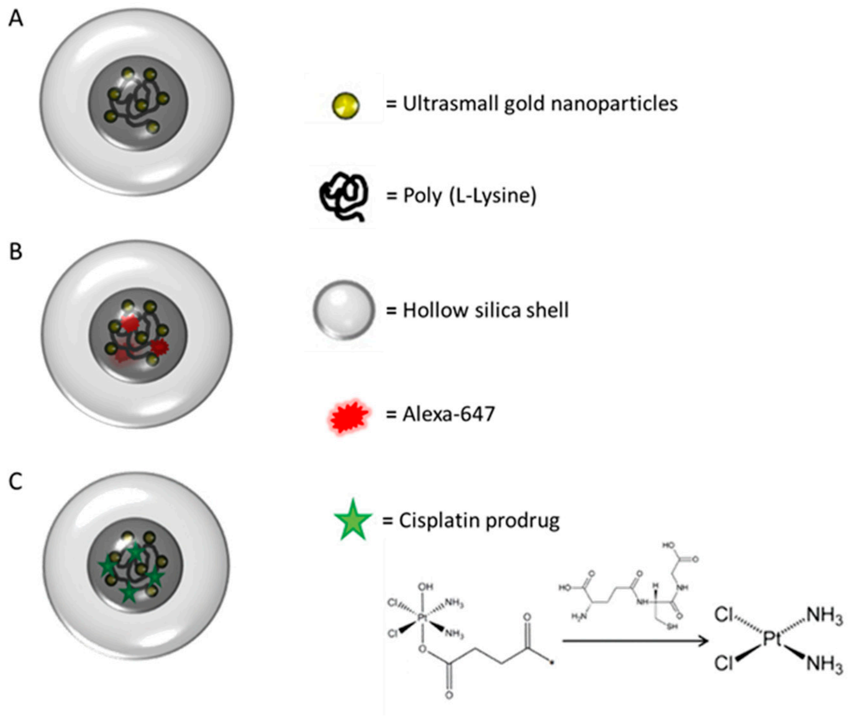

Figure S2. General structure of nano-architectures. **A** Standard gold nanoparticles (NAs). **B** NAs produced using a fluorophore-modified Poly (L-Lysine) with Alexa-647 (NAs-647). **C** Cisplatin loaded nanoparticles (NAs-cisPt).

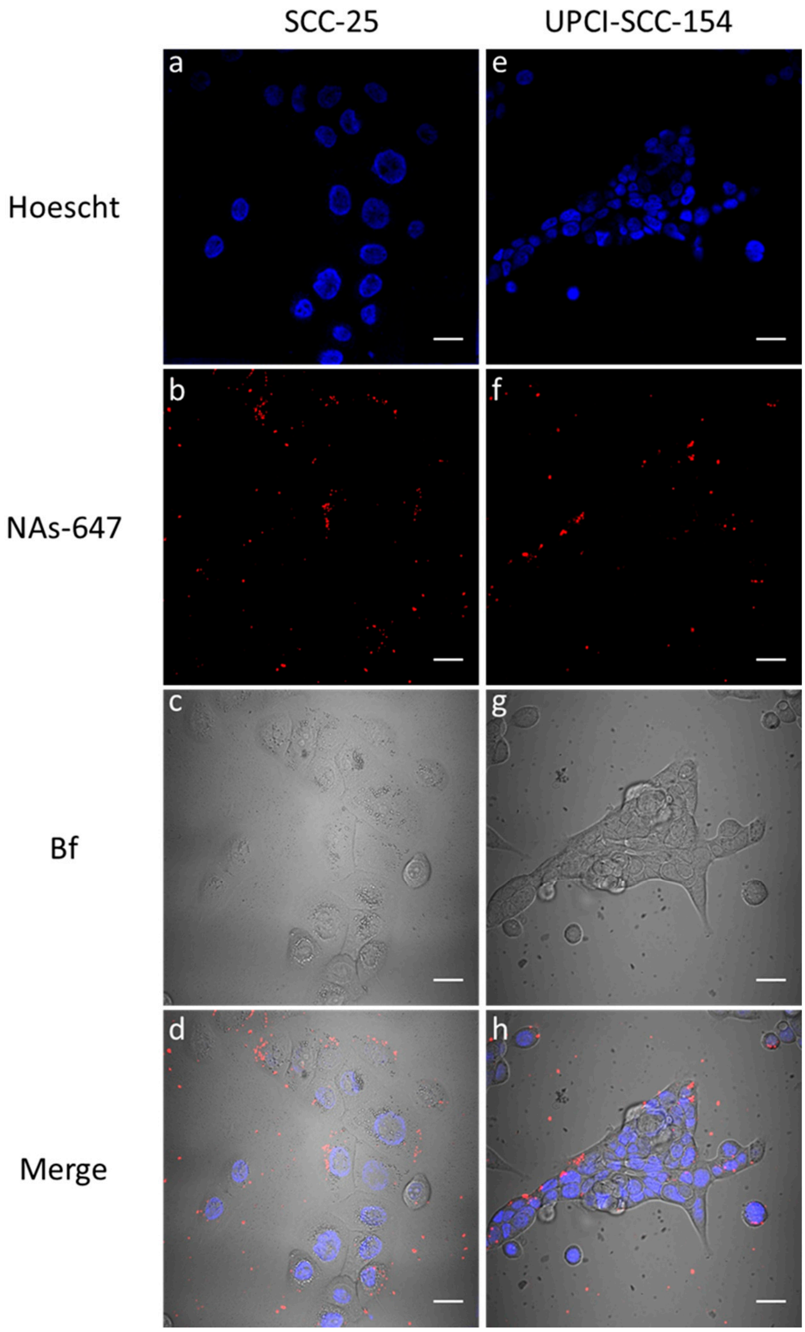

Figure S3. Internalization of NAs-647 in SCC-25 and UPCI:SCC-154 cell lines. Each cell line was treated with a maximum of 30 μg of fluorescently labeled nanoparticles (NAs-647) and incubated for 2h. Internalization was monitored by confocal microscopy. (**a**–**d**) HNSCC-25 and (**e**–**f**) UPCI-SCC-154 treated with NAs-647 (red channel) and Hoechst for nuclei (blue channel). Scale bar: 10 μm.

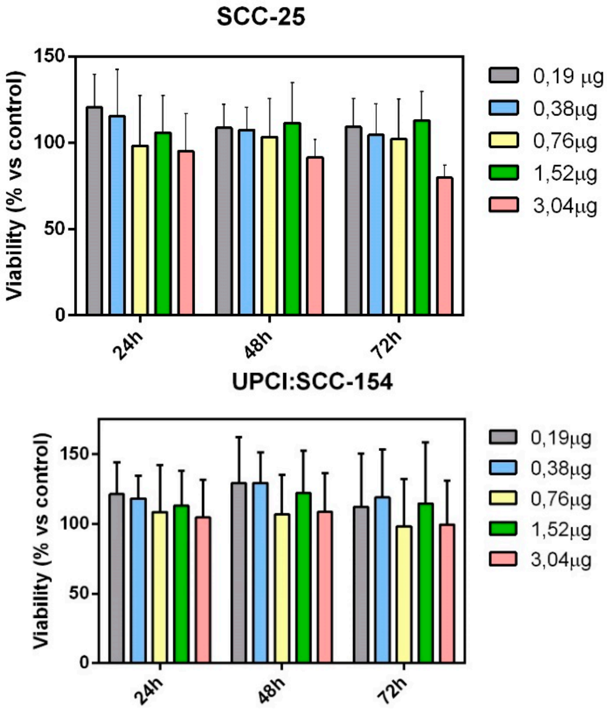

Figure S4. Cytotoxic effect of standard gold nanoparticles (NAs) against SCC-25 and UPCI:SCC-154. Each cell line was treated with increasing concentration of standard nanoparticles corresponding to the amount of gold used with NAs-CisPt. Cells viability were measured during time until 72h after treatment, and related to the viability of control cells treated only with medium. The amount of gold shown in the graph correspond to the actual amount of gold contained in NAs-CisPt used to treat the cells in the previous experiment. Results are the average of three independent experiments and error bars state the standard deviation. Two-way ANOVA Dunnett's test vs. 0,19 µg, no significant statistical differences were found.

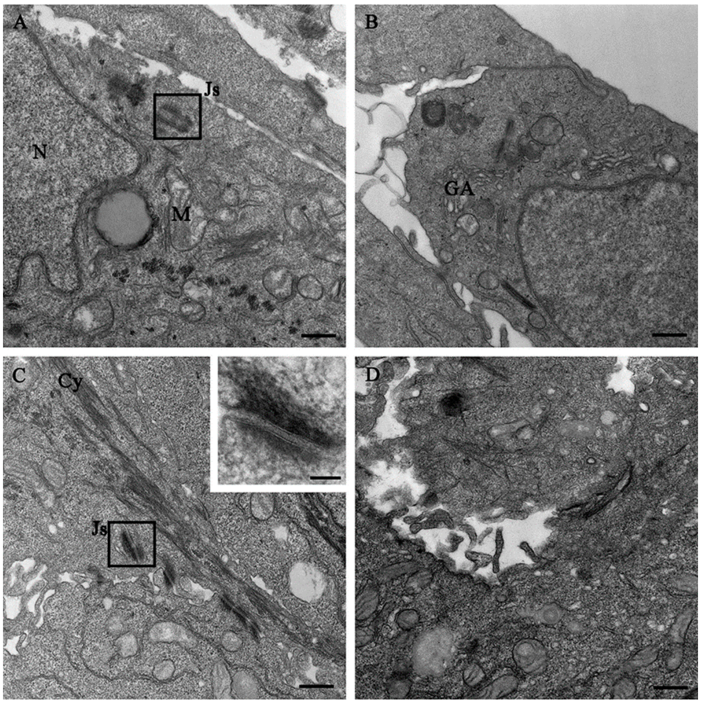

Figure S5. TEM ultrastructural analysis of 2D and 3D models of HNSCCs. (**A**) 2D cell monolayer of SCC-25. (**B**) 2D cell monolayer of UPCI-SCC-154. (**C**) 3D models of SCC-25. (**D**) 3D models of UPCI-SCC-154. Scale bar: 500 nm (inset: 100nm). N = Nuclei; M = Mitochondria; GA = Golgi Apparatus; Cy = cytoskeleton; Js = cell junction.

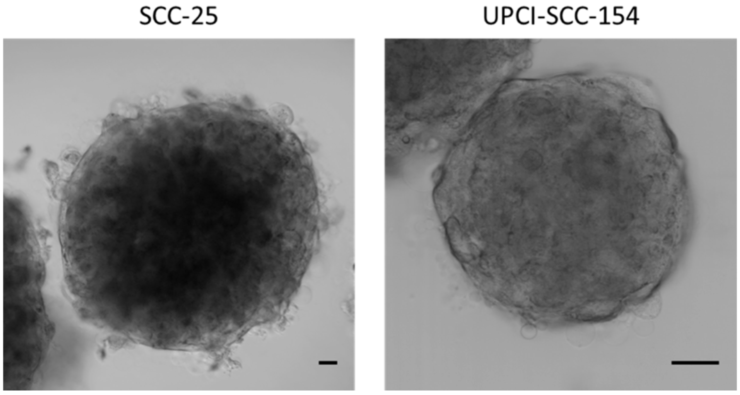

Figure S6. Bright field images of SCC-25 and UPCI-SCC-154 spheroids. Confocal analysis (only bright field) of ±HPV-associated spheroids. Scale bars: SCC-25 = 20 μm, UPCI-SCC-154 = 100 μm.

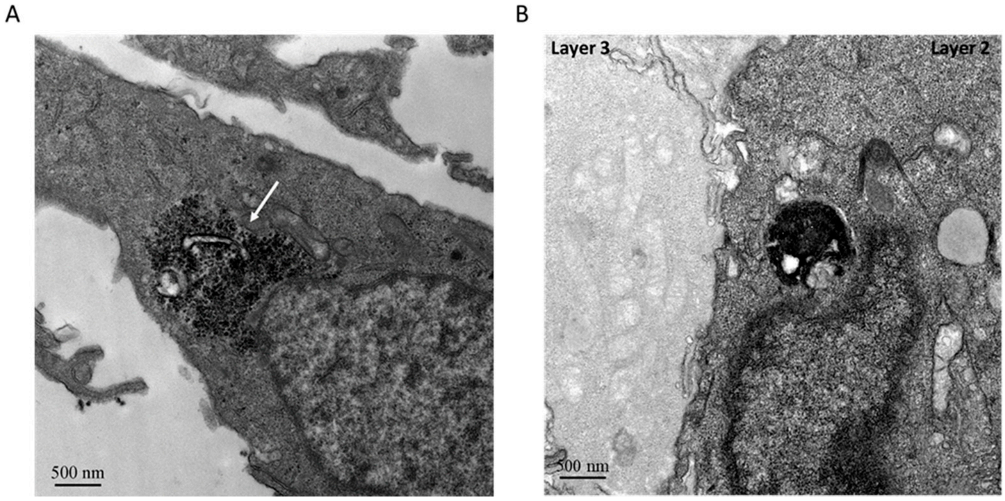

Figure S7. Ultrastructural characterization of 2D and 3D UPCI-SCC-154. TEM characterization of (**A**) 2D monolayer of UPCI-SCC-154 that confirm the presence of the virus inside cells (arrow) and (**B**) a portion of a 3D spheroid of UPCI-SCC-154. Four different layers of cells were identified in these structures. In particular here are shown the layer 2 composed by normal live cells and the layer 3 that were poorly stained by contrast agent and show clearly necrotic features.

Table S1. Size and zeta potential of NAs, NAs-647 and NAs-cisPt.

Variation	NAs	NAs-647	NAs-cisPt
Size (nm) [a,b]	203.1 ± 1.9	207.2 ± 1.4	227.1 ± 0.7
ζ Potential (mV) [a]	−20.6 ± 0.4	-21.3 ± 0.6	−19.6 ± 0.6

[a]Average of three measurements. [b]Calculated from the intensity signal.

Table S2. IC_{50} measurements for SCC-25 and UPCI-SCC-154 measured 24h after treatment with free or nanoparticles-loaded cisplatin.

Variation	IC_{50} after 24 h (μM)	
Cell line	Free cisPt	NAs-cisPt
SCC-25	8,2 ± 0.1	7.5 ± 0,3
UPCI-SCC-154	3.5 ± 0.1	4.4 ± 0,1

Table S3. Viability of SCC-25 spheroids after treatment with NAs-cisPt.

Varitaion	SCC-25 (% vs MEDIUM)							
	0h		24h		48h		72h	
	Viability	Standard deviation	Viability	Standard deviation	Viability	Standard deviation	Viability	Standard deviation
**MEDIUM**	100	33.1	98.4	23.4	100.4	60.8	80.6	20.6
**DMSO (20%)**	11.9	0.3	1.7	0.1	0.3	0.1	0.2	0.1
**NAs-cisPt 8μM**	107.9	28.7	86.9	17.5	70.3	24.9	42.1	29.4
**NAs-cisPt 16μM**	106.9	21.5	95.6	14.6	88.5	12.4	43.1	28.9
**NAs-cisPt 32μM**	101.3	11.8	92.1	19.3	77.8	10.2	39.7	11.1

Table S4. Viability of UPCI-SCC-154 spheroids after treatment with NAs-cisPt.

Variation	UPCI-SCC-154 (% vs MEDIUM)							
	0 h		24 h		48 h		72 h	
	Viability	Standard deviation	Viability	Standard deviation	Viability	Standard deviation	Viability	Standard deviation
**MEDIUM**	100	17.2	83.3	8.3	76.9	9.8	72.5	17.6
**DMSO (20%)**	62.5	6.6	5.3	1.2	7.1	1.6	6.3	2.5
**NAs-cisPt 4μM**	120.4	15.3	76.5	8.6	68.9	8.4	45.6	7.6
**NAs-cisPt 8μM**	107.8	19.4	76.4	9.3	60.1	8.3	37.9	8.5
**NAs-cisPt 16μM**	120.3	19.4	70.6	9.1	60.6	8.8	32.5	8.2

© 2020 by the authors. Licensee MDPI, Basel, Switzerland. This article is an open access article distributed under the terms and conditions of the Creative Commons Attribution (CC BY) license (http://creativecommons.org/licenses/by/4.0/).
